# Clinical characteristics, treatment outcomes and potential novel therapeutic options for patients with neuroendocrine carcinoma of the prostate

**DOI:** 10.18632/oncotarget.26523

**Published:** 2019-01-01

**Authors:** Leonidas Apostolidis, Cathleen Nientiedt, Eva Caroline Winkler, Anne Katrin Berger, Clemens Kratochwil, Annette Kaiser, Anne-Sophie Becker, Dirk Jäger, Markus Hohenfellner, Clemens Hüttenbrink, Sascha Pahernik, Florian A. Distler, Carsten Grüllich

**Affiliations:** ^1^ Department of Medical Oncology, National Center for Tumor Diseases, University Hospital Heidelberg, Heidelberg, Germany; ^2^ Department of Nuclear Medicine, University Hospital Heidelberg, Heidelberg, Germany; ^3^ Institute of Pathology, Klinikum Nuremberg, Paracelsus Medical University, Nuremberg, Germany; ^4^ Department of Urology, University Hospital Heidelberg, Heidelberg, Germany; ^5^ Department of Urology, Klinikum Nuremberg, Paracelsus Medical University, Nuremberg, Germany

**Keywords:** neuroendocrine carcinoma, neuroendocrine tumor, carcinoid, prostate, chemotherapy

## Abstract

**Background:**

Neuroendocrine carcinomas of the prostate (NEPCs) are rare tumors with poor prognosis. While platinum and etoposide-based chemotherapy regimens (PE) are commonly applied in first-line for advanced disease, evidence for second-line therapy and beyond is very limited.

**Methods:**

Retrospective analysis of all patients with NEPCs including mixed differentiation with adenocarcinoma component and well differentiated neuroendocrine tumors (NETs, carcinoids) at two high-volume oncological centers between 12/2000 and 11/2017.

**Results:**

Of 46 identified patients 39.1 % had a prior diagnosis of prostatic adenocarcinoma only, 43.5 % had a mixed differentiation at NEPC diagnosis, 67.4 % developed visceral metastases, 10.9 % showed paraneoplastic syndromes. Overall survival (OS) from NEPC diagnosis was 15.5 months, and significantly shorter in patients with a prior prostatic adenocarcinoma (5.4 vs. 32.7 months, p=0.005). 34 patients received palliative first-line systemic therapy with a median progression-free survival (PFS) of 6.6 months, mostly PE. Overall response rate (ORR) for PE was 48.1 %. 19 patients received second-line therapy, mostly with poor responses. Active regimens were topotecan (1 PR, 3 PD), enzalutamide (1 SD), abiraterone (1 SD), FOLFIRI (1 SD), and ipilimumab+nivolumab (1 PR). One patient with prostatic carcinoid was sequentially treated with octreotide, peptide receptor radionuclide therapy and everolimus, and survived for over 9 years.

**Conclusions:**

EP in first-line shows notable ORR, however limited PFS. For second-line therapy, topotecan, FOLFIRI, enzalutamide, abiraterone and immune checkpoint blockade are treatment options. Prostatic carcinoids can be treated in analogy to well differentiated gastrointestinal NETs.

## INTRODUCTION

Neuroendocrine Carcinoma (NEC) of the prostate (NEPC) is considered a rare tumor entity with a rising incidence [1]. Compared to conventional adenocarcinoma, NEPC is characterized by an aggressive tumor biology with loss of PSA secretion, unresponsiveness to androgen deprivation therapy (ADT), development of visceral metastases and limited prognosis [2]. NEPC can feature an adenocarcinoma component, resulting in a mixed differentiation. Besides NEPC arising de novo, more commonly they are described in the context of castration-resistant prostate cancer after ADT.

Compared to adenocarcinoma, prognosis of NEPC is poor with survival ranging from 7 to 10 months. Due to the rarity of the disease, the optimal treatment strategy is up to debate. Like in NECs of other organ systems, platinum-based chemotherapy regimens are commonly applied in first-line for advanced disease [3–9]. Despite encouraging response rates in several phase II studies, progression-free (PFS) and overall survival (OS) are short. Clinical-grade evidence for systemic treatment options in second-line and beyond is extremely limited [3, 10, 11].

On the other hand, well differentiated Neuroendocrine Tumors (NETs, carcinoids) of the prostate are even rarer than highly proliferative NEPCs with only few reported cases in the literature [12, 13]. While the biology is more indolent than in poorly differentiated NEPC, response to chemotherapy is generally poor [14]. Treatment with somatostatin analogues, targeted agents and peptide receptor radionuclide therapy (PRRT) is considered standard of care for NETs of the gastrointestinal tract [15] but has not been evaluated in prostatic NETs (carcinoids) so far.

The aim of our study was to investigate the clinical characteristics and treatment outcome of patients with NEPC.

## RESULTS

### Patient characteristics

A total of 46 male patients with a median age of 69 years were identified (Table 1). Median follow-up was 61.0 months. 39.1 % of patients had a prior diagnosis of prostatic adenocarcinoma only, the median interval between adenocarcinoma and NEPC diagnosis was 28.2 months (range 1.0-123.8). 20 men (43.5 %) showed a mixed differentiation at NEPC diagnosis. Small cell histology was documented in 45.7 % of patients. Median proliferation rate (Ki67) was 80 %. 1 Patient with non-small cell histology was classified as carcinoid (well differentiated NET) with a Ki67 of only 1 %. 8 patients (17.4 %) were diagnosed in a localized stage. The remaining 82.6 % of patients (75.0 % with de-novo NEPC, 94.4 % with prior adenocarcinoma) were diagnosed with synchronous metastatic NEPC, with 67.4 % developing visceral (i.e. non-bone, non-lymphatic) metastases and 65.2% developing bone metastases during the course of the disease. Median tumor marker levels were 3.2 x ULN (upper limit normal) for PSA, 6.1 x ULN (i.e. 518.0 ng/ml) for chromogranin A and 7.6 x ULN (i.e. 128.5 ng/ml) for NSE (neuron-specific enolase). 10.9 % of patients showed paraneoplastic syndromes, including 1 case of paraneoplastic neuropathy, 1 case of disseminated intravasal coagulation, 1 case of ectopic ACTH (adrenocorticotropic hormone) production and 2 cases of SIADH (syndrome of inappropriate antidiuretic hormone secretion).

**Table 1:** Patient characteristics at NEPC diagnosis

			Number of patients N=46	%
Age [years]	Median	69		
	Range	51-82		
Stage	Localized		8	17.4
	Metastatic		38	82.6
Metastatic sites	Lymph nodes		34	73.9
	Bone		30	65.2
	Liver		20	43.5
	Lung		14	30.4
	Brain		8	17.4
	Pleura		3	6.5
	Adrenal gland		2	4.3
	Peritoneum		3	6.5
	Other		4	8.7
Paraneoplastic	SIADH		2	4.3
syndromes	Ectopic ACTH production		1	2.2
	Neuropathy		1	2.2
	DIC		1	2.2
Ki67 [%]	Median	90		
	Range	1-100		
	< 55		9	19.6
	≥ 55		25	54.3
Histology	Small cell		21	45.7
	Non small cell		25	54.3
	Mixed differentiation		20	43.5
	Carcinoid		1	2.2
Prior prostatic adenocarcinoma			18	39.1
Tumor markers	PSA > ULN		26	56.5
	PSA ≤ ULN		6	13.0
	NSE > ULN		21	45.7
	NSE ≤ ULN		3	6.5
	CgA > ULN		12	26.1
	CgA ≤ ULN		3	6.5
	LDH > ULN		13	28.3
	LDH ≤ ULN		18	39.1
Therapy prior to NEPC diagnosis	Surgery of primary		19	41.3
	Radiotherapy of primary		9	19.6
	Androgen deprivation therapy		17	27.0
	Abiraterone		4	8.7
	Enzalutamide		3	6.5
	Docetaxel		6	13.0
	Cabazitaxel		2	4.3
	PSMA radionuclide therapy		1	2.2

### Survival

**Figure 1:**
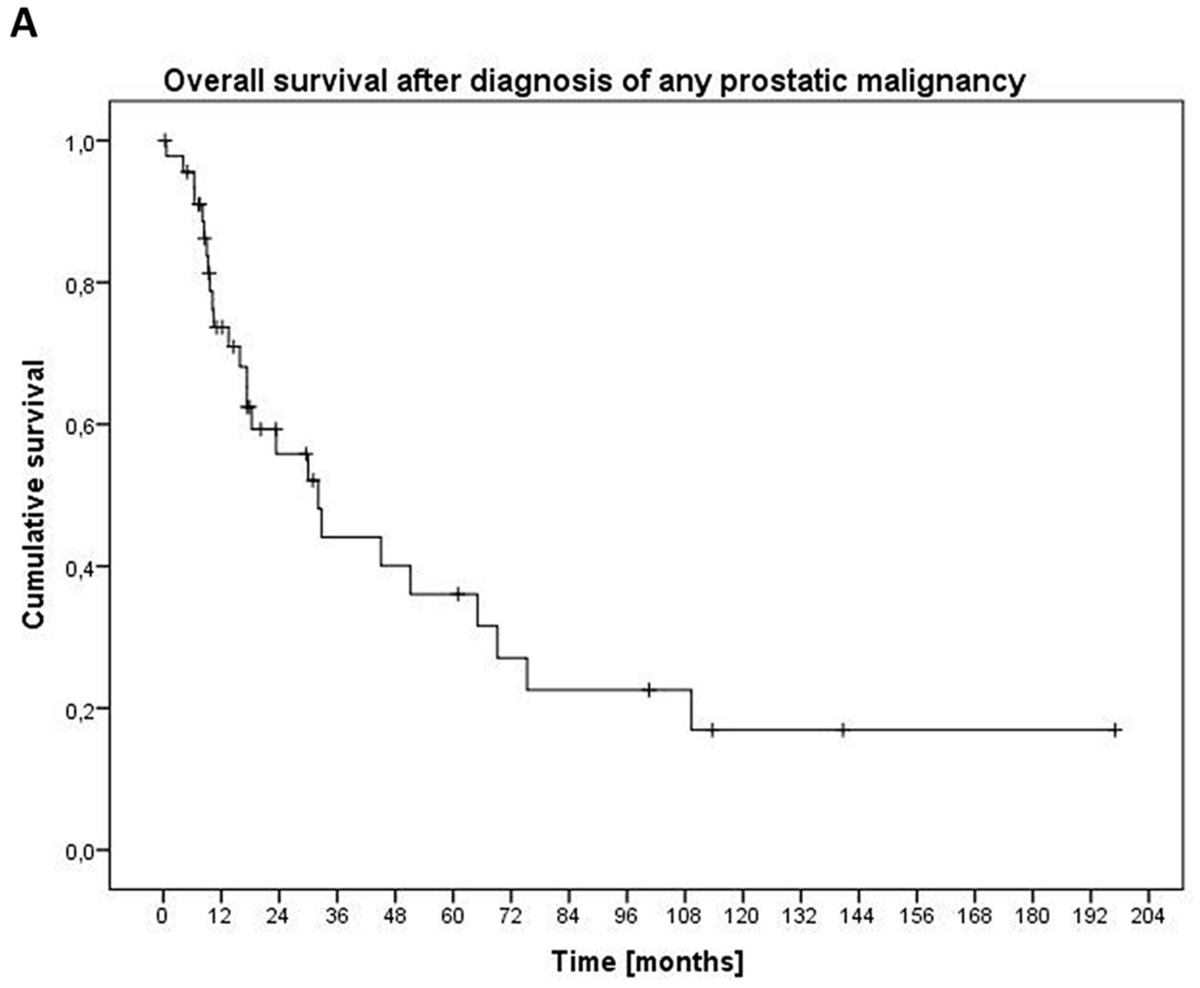

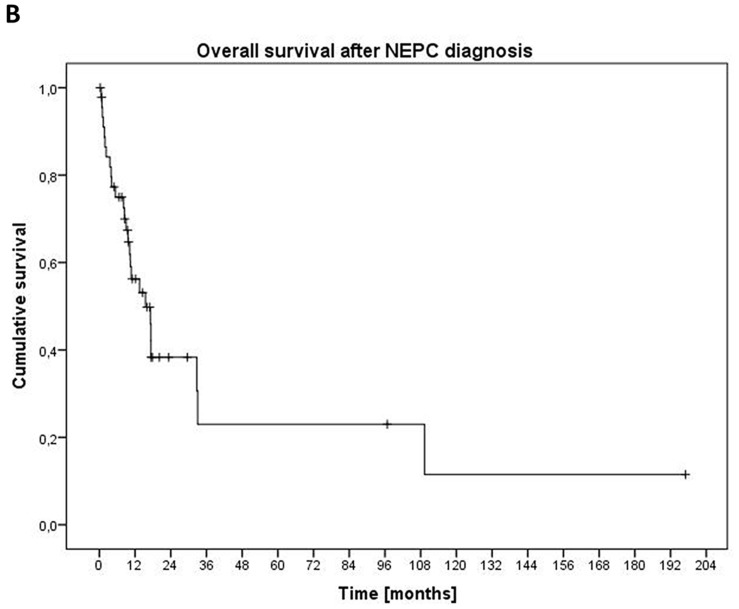
Overall survival from diagnosis of any prostatic malignancy **(A)** and from diagnosis of NEPC **(B)**.

Median OS from the timepoint of diagnosis of any prostatic malignancy was 32.1 months, from the timepoint of NEPC diagnosis it was 15.5 months (Figure 1) with 6-month survival rate of 0.76 (95 % CI 0.63-0.87) and 12-month survival rate of 0.57 (95 % CI 0.41-0.73). In a univariate analysis of OS in different subgroups, patients with previous history of prostatic adenocarcinoma had a significantly worse prognosis (5.4 vs. 32.7 months, p=0.005) (Table 2, see Supplementary Table 1 for 6-month and 12-month survival proportion estimates). There was no significant difference in OS of patients with mixed vs. purely neuroendocrine differentiation. Patients with elevated levels of lactate dehydrogenase (LDH) showed a strong trend towards a shortened OS of 5.4 vs. 17.3 months (p=0.064). For patients with metastatic disease not receiving any palliative systemic therapy for NEPC, median OS was 3.9 months.

**Table 2:** Overall survival from the timepoint of NEPC diagnosis in different subgroups

		Median OS [months]	p
Histology	Small cell	15.5	0.828
	Non-small cell	17.1	
Mixed differentiation	Yes	15.5	0.970
	No	17.3	
Prior adenocarcinoma	Yes	5.4	0.005
	No	32.7	
Ki67	≥ 55 %	10.4	0.325
	< 55 %	17.1	
PSA	> ULN	10.7	0.719
	≤ ULN	33.1	
NSE	> ULN	9.6	0.105
	≤ ULN	NR	
CgA	> ULN	15.5	0.330
	≤ ULN	9.6	
LDH	> ULN	5.4	0.064
	≤ ULN	17.3	
Stage	Localized	32.7	0.411
	Metastatic	15.5	
Visceral metastases	Yes	13.5	0.166
	No	NR	
Palliative systemic therapy	Yes	17.4	0.192
	No	3.9	

### Efficacy of first-line therapy

**Figure 2:**
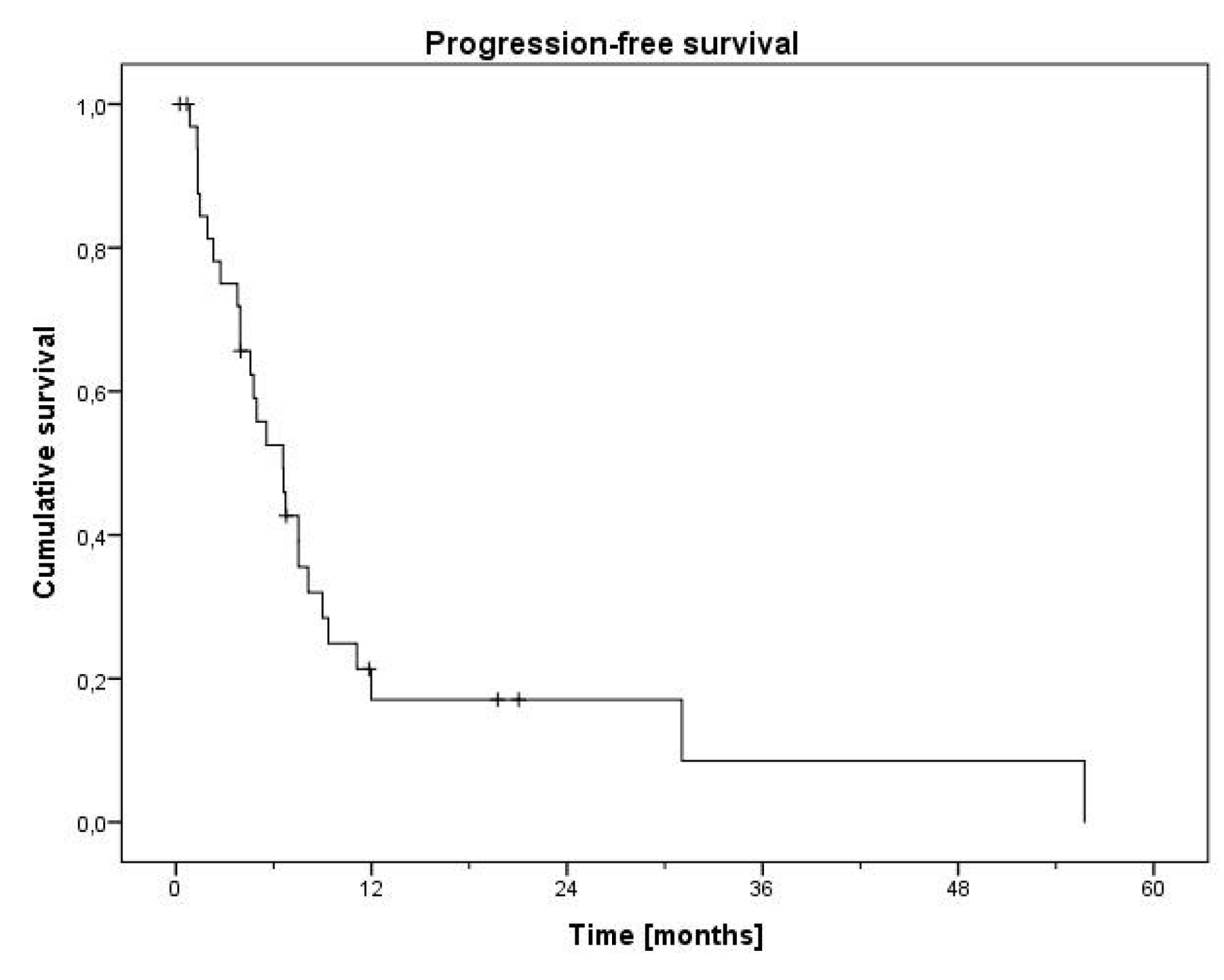
Progression-free survival of first-line therapy

**Figure 3:**
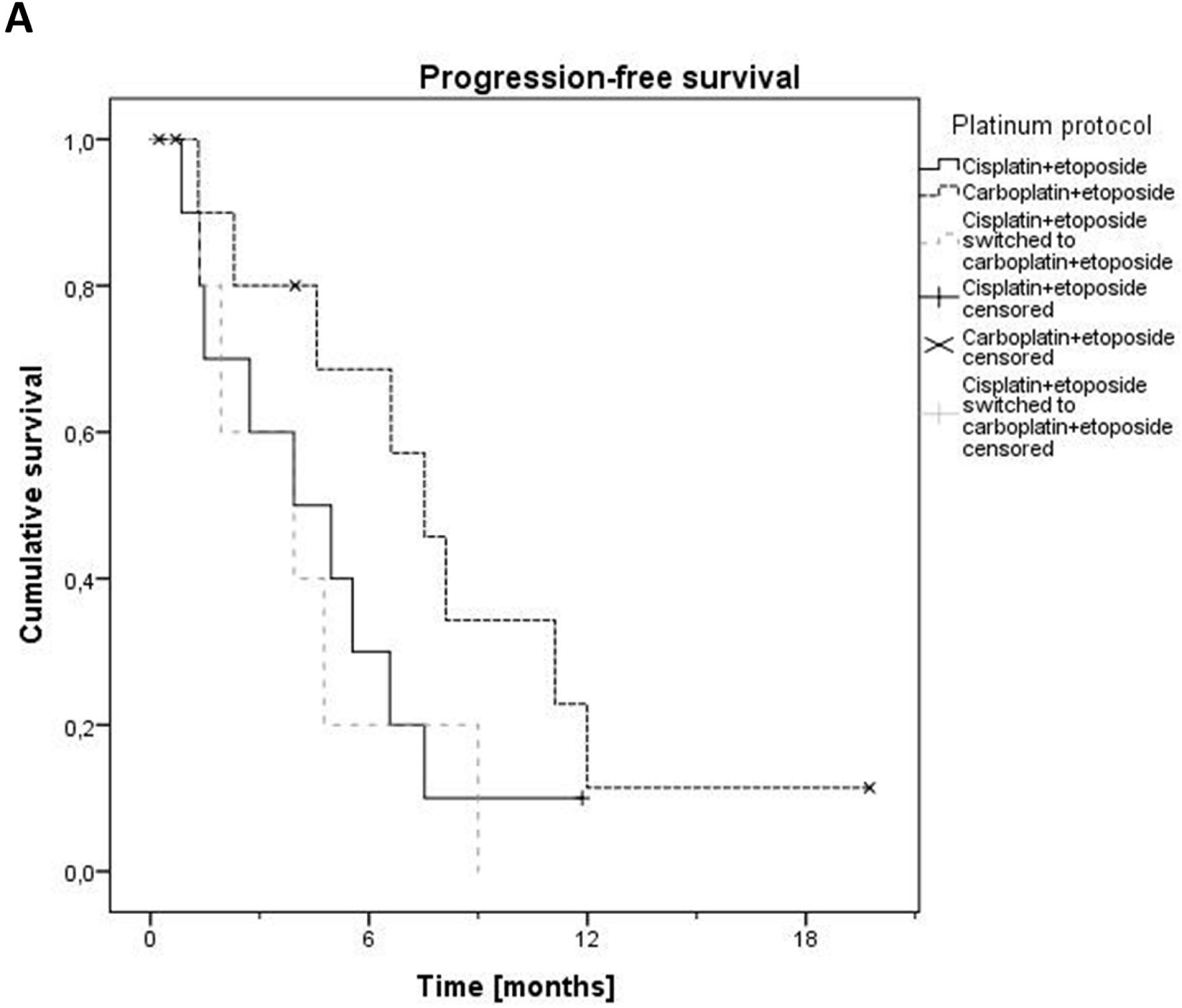

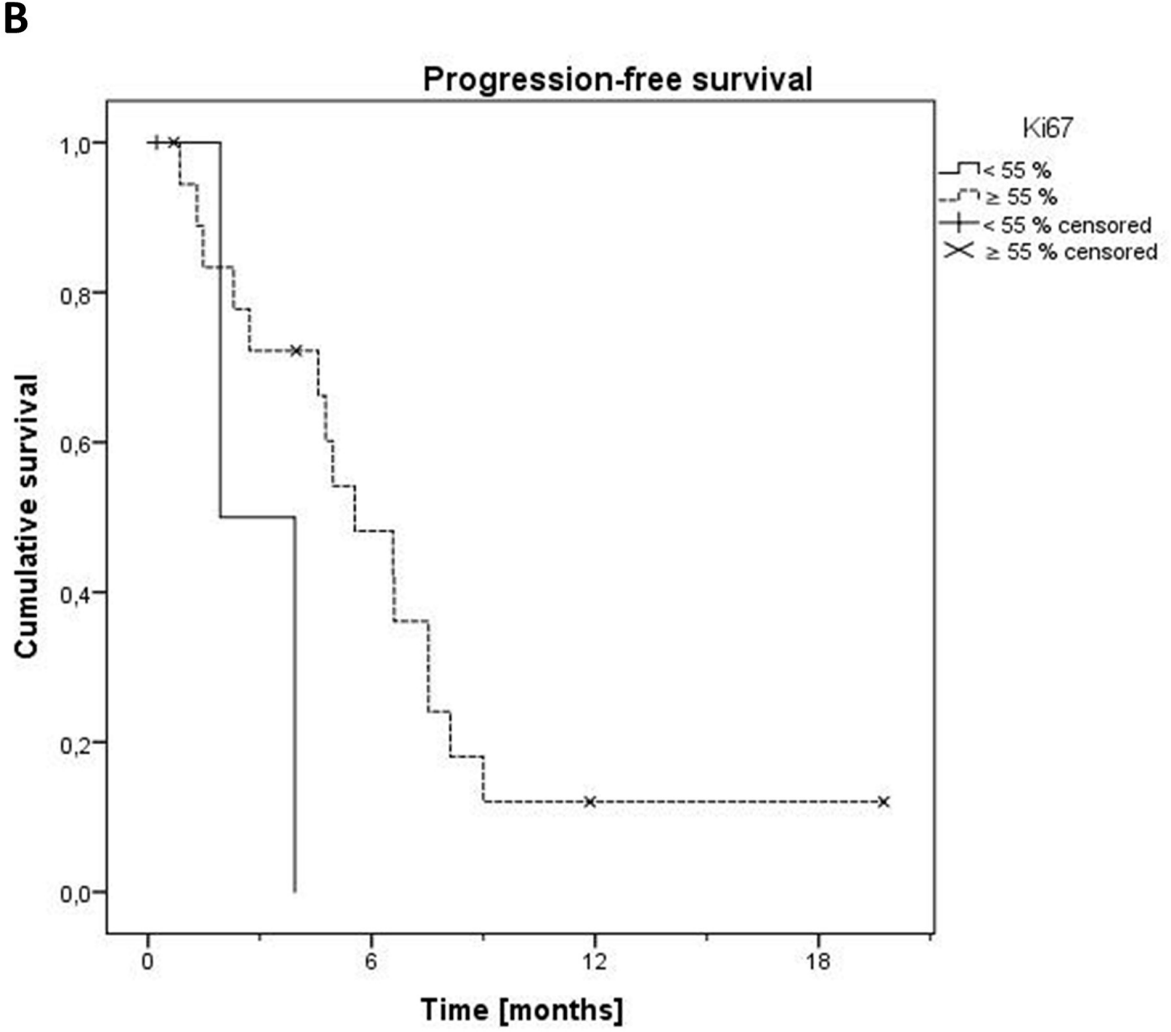
Progression-free survival for platinum and etoposide regarding type of platinum **(A)** and Ki67 (≥ 55 % vs. < 55 %) **(B)**.

34 patients received palliative first-line systemic therapy, mostly platinum and etoposide (PE) (n=27). A median of 5 cycles of PE was administered (range 1-9). Median PFS was 6.6 months (Figure 2). Overall response rate (ORR) for PE was 48.1 % (1 complete response [CR], 12 partial responses [PR], 1 stable disease [SD]). ORR was higher in patients with small cell vs. non-small cell histology (56.3 % vs. 36.4 %), however similar in patients with mixed vs. pure neuroendocrine differentiation (45.5 vs. 50.0 %). Of the patients receiving PE, 10 patients were treated with cisplatin and 12 patients received carboplatin. 5 patients switched from cisplatin to carboplatin because of toxicity after a median of 2 cycles. Patients primarily treated with carboplatin showed a trend towards a prolonged PFS of 7.5 months vs. those receiving cisplatin (3.9 months, p=0.114) (Figure 3A). PFS for patients who switched to carboplatin was similar to those who only received cisplatin (3.9 months). Only 3 Patients with proliferation rate (Ki67) of < 55 % were treated with PE. They showed a strong trend towards a lower PFS of 1.9 months vs. patients with a Ki67 ≥ 55 % (5.6 months, p=0.071, Figure 3B). Of patients who received another first-line therapy than PE, only 6 were evaluable for response, 1 PD with docetaxel, 1 SD and 1 PR with FOLFIRI, 1 SD with abiraterone, 1 SD with enzalutamide. The sixth patient was treated with the somatostatin analogue octreotide for his well differentiated NET (carcinoid), which resulted in a disease stabilization for 31.1 months.

### Second-line therapy and beyond

19 patients received second-line therapy, mostly with poor response rates (Table 3).

**Table 3:** Overview of second-line therapies

	Total	CR	PR	SD	PD	NE	median DoR [months] (for CR, PR, SD)
PE	5		3	1		1	8.0
Topotecan	5		1		3	1	5.9
FOLFIRI	1			1			8.4
FOLFOX	1				1		
Docetaxel	1					1	
Enzalutamide	1			1			8.1
Abiraterone	1					1	
Everolimus	1				1		
PRRT	1		1				37.5
SIRT	1				1		
Ipilimumab+nivolumab	1		1				7.1

**Figure 4:**
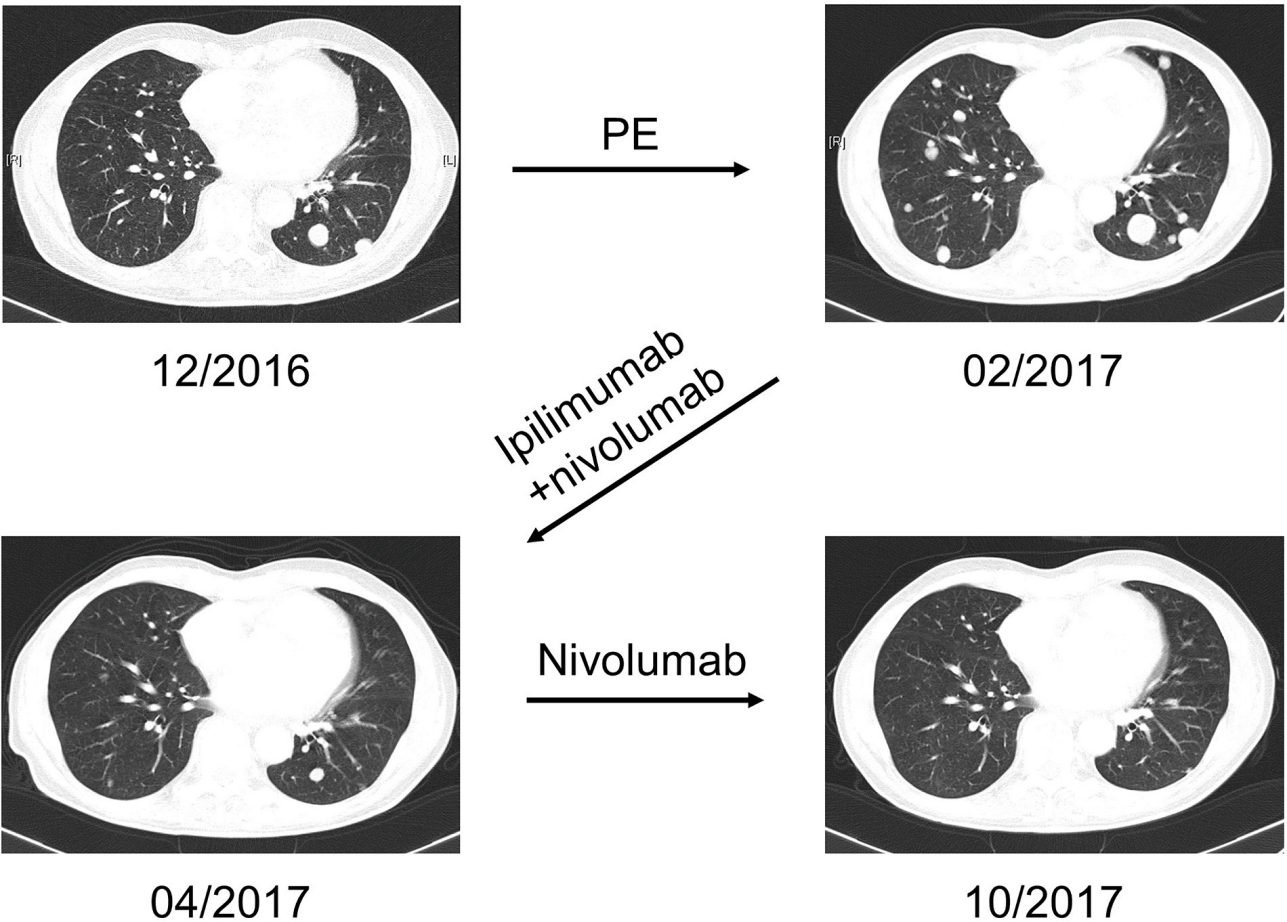
Case example of sustained partial remission to immune checkpoint blockade CT scans of a 70-year old patient with small cell NEPC with minor adenocarcinoma component, Ki67 85 %. After direct progression to PE, the patient was treated with 4 cycles of dual immune checkpoint blockade with ipilimumab+nivolumab, following nivolumab maintenance therapy. He showed a very good PR for more than 6 months.

Beside PE, regimens with notable activity were topotecan, enzalutamide, and FOLFIRI. Most notably, 1 patient primary refractory to PE showed a very good PR under dual immune checkpoint blockade with ipilimumab+nivolumab for more than 6 months (Figure 4). The patient with the prostatic carcinoid showed prolonged disease stabilization for 37.9 months under PRRT targeting the somatostatin receptor.

Third-line therapy was applied to 6 patients only. Of those, only the carcinoid patient showed disease stabilization under everolimus, 1 patient showed a CR to re-exposition with PE. The patient treated with ipilimumab+nivolumab showed a progressive bone metastasis after 7.1 months under nivolumab maintenance therapy. Upon reinduction with ipilimumab, immunotherapy had to be stopped due to a grade 3 autoimmune colitis after the first cycle. After palliative bone radiotherapy, he received enzalutamide which resulted in short-term disease stabilization. The other 3 patients showed PD (1x FOLFIRI, 1x docetaxel, 1x carboplatin+paclitaxel).

**Figure 5:**
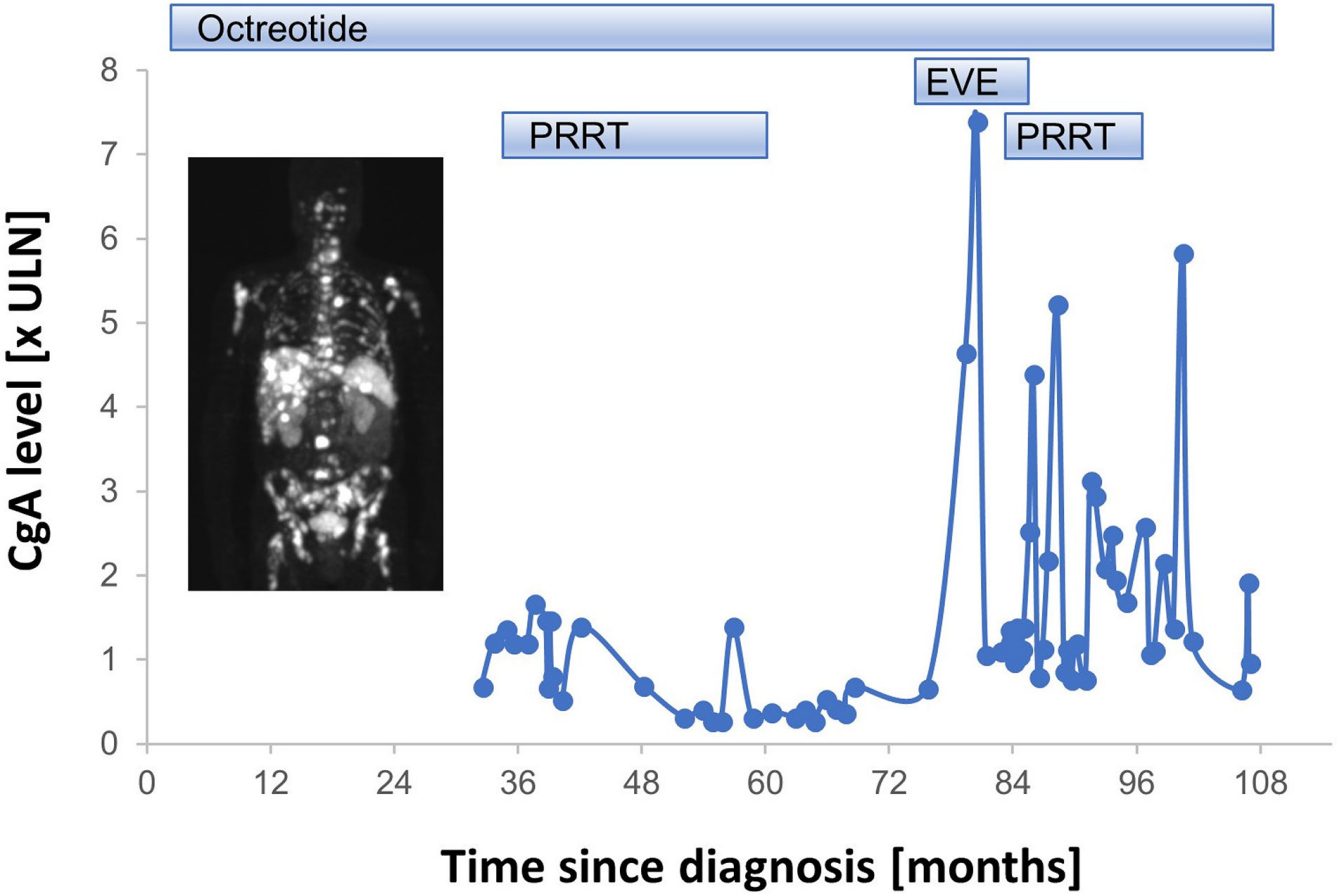
Overview of systemic therapy sequence and tumor marker chromogranin A (CgA) of a patient with metastatic prostatic well differentiated NET (carcinoid) Insert: representative DOTATOC-PET/CT scan showing somatostatin receptor positive lesions. EVE: everolimus; PRRT: peptide receptor radionuclide therapy; ULN: upper limit normal.

The carcinoid patient received a salvage PRRT with an alpha emitter, the therapy had to be discontinued due to progressive bone marrow carcinosis and increasing bone marrow insufficiency. Finally, he died from progressive disease 109.3 months after NET diagnosis. The sequence of systemic therapies and chromogranin A levels are summarized in Figure 5.

## DISCUSSION

The clinical characteristics of our NEPC patient cohort are in line with previous reports, including the frequent presence of visceral metastases and paraneoplastic syndromes, as well as elevated neuroendocrine tumor markers (chromogranin A, NSE) [2, 16]. The worse prognosis of patients with a prior history of adenocarcinoma can be most likely explained by the several lines of systemic therapies those patients have already received for adenocarcinoma when they are diagnosed with NEPC, leading to a selection of a more resistant disease. On the other hand, the similar behavior of patients with pure neuroendocrine and mixed adeno-neuroendocrine differentiation (regarding OS and response to first-line therapy) might indicate that the neuroendocrine component seems to be the main prognostic driver of the disease regardless of a coexisting adenocarcinoma component.

Platinum and etoposide-based chemotherapy is considered standard of care for high grade NEC in different locations, including lung and gastrointestinal tract [17]. Several phase II trials have examined platinum-based combination treatments in NEPC [3–9]. The trials are difficult to compare since in some histological proof for NEPC was mandatory, whereas others recruited also patients with only clinical features suggestive for NEPC. However, response rates and survival were not considerably higher in trials examining a platinum-based combination therapy employing 3 agents compared to those with 2 agents. In general, both cisplatin and carboplatin-based chemotherapy regimens are considered equally effective in NEC [17]. However, in our study there was a slight trend for a prolonged PFS with carboplatin. This may be due to a selection bias in our small retrospective cohort. Additionally, the toxicity profile of cisplatin might contribute to more frequent treatment interruptions and delays in an elderly patient population. Patients with a lower Ki67 of < 55 % showed a strong trend for a shortened PFS under PE, this difference failed to reach significance most likely due to the small patient numbers in this group. This phenomenon has already been described in several retrospective analyses of NEC of the gastrointestinal tract [18–20], and finally led to a newly defined tumor entity of well differentiated neuroendocrine tumors grade 3 (NET G3) which has been officially introduced with the World Health Organization Classification of Tumors of Endocrine Organs of 2017. Current treatment guidelines recommend alternative treatment protocols to PE for NET G3 which have shown notable activity in second-line after PE failure [17, 21, 22], but have not been evaluated in first-line situation yet. The entity of NET G3 has not been established in NEPC so far.

Regarding second-line therapy or alternatives to platinum-based chemotherapy, evidence for NEPC is very scarce. One of the above-mentioned studies applied second-line therapy with cisplatin and etoposide after progression to the first-line treatment with carboplatin and docetaxel [3]. Combination therapy of doxorubicin, cyclophosphamide and vincristine [9] as well as amrubicin monotherapy [23] showed some activity in small case series. Molecular alterations in NEPCs have been extensively studied. Besides alterations common to prostatic adenocarcinoma like TMPRSS2-ERG fusion and alterations in DNA damage repair proteins (e.g. BRCA1, BRCA2, FANCA), several genes have been identified specific for neuroendocrine transdifferentiation, like TP53, RB1, AURKA (Aurora Kinase A), MYCN, and MTOR [24–30]. A comprehensive genomic characterization of treatment related NEPC, reported further transcriptomic markers like PDX1, EZH2, BRN2, FOXA2 and ASCL1 [31]. Recently, a phase II trial with the AURKA inhibitor alisertib was presented with a median PFS of 8.7 weeks [11]. Although it failed to meet its primary endpoint with a 6-month PFS of only 12.6 %, 3 of 59 patients showed exceptional remissions or disease stabilizations. In a preliminary retrospective analysis of 7 patients treated with the MTOR inhibitor everolimus a decrease in tumor markers was noted in 5 patients [10]. In our analysis, the single patient receiving everolimus for highly proliferative NEPC showed PD; however, a disease stabilization and tumor marker decrease was noted in the patient with well differentiated NET (carcinoid).

Regarding the limited evidence, second-line treatments recommended for non-prostatic NEC can also be considered an option for NEPC. Topotecan is a standard of care therapy for small cell NEC of the lung [32]. However, the activity of topotecan in extrapulmonary NEC is very limited [33, 34]. 1 of 4 patients treated with topotecan showed a PR in our analysis. FOLFIRI has been studied in NEC of the gastrointestinal tract [35]. In our study, it showed disease stabilization in first-line, as well as in pretreated patients.

Immune checkpoint blockade has shown promising activity in multiple types of NEC, including small cell lung cancer [36], Merkel cell carcinoma [37, 38], as well as in NEC of the pancreas [39] and cervix [40]. Here we report an extremely good response of NEPC to combined immune checkpoint blockade with ipilimumab and nivolumab.

NEPC is considered to be refractory to ADT [41] and a recent study-cohort showed a high resistance to modern androgen receptor–targeting therapies of up to 73% [31]. However, 3 of our patients showed a short disease stabilization under enzalutamide and abiraterone. This is in line with several recent reports for large-cell NEPC which have shown expression of androgen receptor as well as sustained responses to conventional ADT [42, 43]. This contributes to the evidence that some androgen dependency might still exist in selected cases, but especially by considering the background of the lineage plasticity model [26], the precise circumstances of the androgen receptor for developing and/or treating a NEPC demands further exploration.

Finally, we reported the first patient with a metastatic well differentiated NET (carcinoid) of the prostate receiving several lines of systemic therapy. He was treated in analogy to NETs of the gastrointestinal tract with octreotide [44], PRRT [45] and everolimus [46]. All systemic therapies were effective in disease stabilization, the patient survived for more than 9 years. Notably, also well differentiated NET have been rarely reported to develop from prostatic adenocarcinoma under ADT [47]; furthermore, somatostatin receptor expression can also be detected in high grade NEPC, indicating that PRRT might be a possible treatment option not only for well differentiated NETs [48].

Our study has several limitations due to its retrospective nature. However, it provides important evidence for potentially active therapeutic regimens in NEPC. PFS seems to be prolonged for carboplatin vs. cisplatin-based regimens, and activity of PE seems lower in patients with a Ki67 < 55 %. The limited prognosis especially of patients with prior history of prostatic adenocarcinoma should be taken into consideration regarding the further clinical decision making for these patients. Most notably, efficacy of FOLFIRI, novel antiandrogens and immune checkpoint blockade should be further evaluated prospectively. Responses should be correlated to clinical parameters as well biomarkers, to further optimize the treatment of this rare disease. Furthermore, the even rarer well differentiated NETs of the prostate (carcinoids) can be treated very successfully in analogy to NETs of the gastrointestinal tract with somatostatin analogues, PRRT and everolimus.

## PATIENTS AND METHODS

We retrospectively reviewed the medical records of all patients with histologically proven NEPC who were treated at the National Center for Tumor Diseases, University Hospital Heidelberg as well as at the Department of Urology, Klinikum Nuremberg, Paracelsus Medical University between 12/2000 and 11/2017. NEPCs with mixed differentiation including an adenocarcinoma component were included at the analysis, as well as well differentiated NETs (carcinoids).

The duration of each therapy as well as the response according to RECIST criteria were recorded, PFS and OS were calculated. PFS was defined as the time span between the start of the respective therapy and the date of progression or death due to any cause. OS was defined on the one hand as the time length between diagnosis of any prostatic malignancy and the date of death from any cause, on the other hand as the time length between NEPC diagnosis and the date of death from any cause.

Statistical analysis was carried out using SPSS™ for Windows™ Software V22.0 (SPSS, Chicago, IL, USA). Survival analysis was calculated using the Kaplan-Meier method, and differences in survival were analyzed using the log-rank test. A p-value of < 0.05 was considered significant.

The trial was approved by the institutional research ethics committee (approval S-428/2014).

## SUPPLEMENTARY MATERIALS TABLE



## Abbreviations

ACTH: adrenocorticotropic hormone
ADT: androgen deprivation therapy
AURKA: Aurora Kinase A
CgA: chromogranin A
CI: confidence interval
CR: complete response
DIC: disseminated intravascular coagulation
DoR: duration of response
LDH: lactate dehydrogenase
NE: not evaluated
NEC: neuroendocrine carcinoma
NEPC: neuroendocrine carcinoma of the prostate
NET: neuroendocrine tumor
NR: not reached
NSE: neuron-specific enolase
ORR: overall response rate
OS: overall survival
PD: progressive disease
PE: platinum+etoposide
PFS: progression-free survival
PR: partial response
PRRT: peptide receptor radionuclide therapy
PSMA: prostate-specific membrane antigen
SD: stable disease
SIADH: syndrome of inappropriate antidiuretic hormone secretion
SIRT: selective internal radiotherapy
ULN: upper limit normal,
