# Supplementary material for: Clinical characteristics, treatment outcomes and potential novel therapeutic options for patients with neuroendocrine carcinoma of the prostate
Source: Oncotarget. 2019 Jan 1;10(1):17–29. doi: 10.18632/oncotarget.26523 (PMC6343754; doi:10.18632/oncotarget.26523)
Supplement: Supplementary file 1 [file oncotarget-10-17-s001.pdf]

Clinical characteristics, treatment outcomes and potential novel therapeutic options for patients with neuroendocrine carcinoma of the prostate

SUPPLEMENTARY MATERIALS

Supplementary Table 1: Overall survival proportion estimates for different subgroups

		6-month survival (95 % CI)	12-month survival (95 % CI)
Histology	Small cell	0.85 (0.69 - 1.00)	0.61 (0.37 - 0.85)
	Non-small cell	0.67 (0.49 - 0.85)	0.54 (0.34 - 0.74)
Mixed differentiation	Yes	0.85 (0.69 - 1.00)	0.62 (0.40 - 0.84)
	No	0.68 (0.50 - 0.86)	0.53 (0.33 - 0.73)
Prior adenocarcinoma	Yes	0.50 (0.26 - 0.74)	0.38 (0.14 - 0.62)
	No	0.92 (0.80 - 1.00)	0.69 (0.49 - 0.89)
Ki67	≥ 55 %	0.71 (0.53 - 0.89)	0.43 (0.23 - 0.63)
	< 55 %	0.76 (0.47 - 1.00)	0.63 (0.30 - 0.96)
PSA	> ULN	0.69 (0.51 - 0.87)	0.51 (0.31 - 0.71)
	≤ ULN	0.67 (0.30 - 1.00)	0.67 (0.30 - 1.00)
NSE	> ULN	0.66 (0.46 - 0.86)	0.44 (0.22 - 0.66)
	≤ ULN	1.00 (1.00 - 1.00)	1.00 (1.00 - 1.00)
CgA	> ULN	0.65 (0.38 - 0.92)	0.55 (0.26 - 0.84)
	≤ ULN	1.00 (1.00 - 1.00)	0.60 (0.00 - 1.00)
LDH	> ULN	0.50 (0.23 - 0.77)	0.28 (0.01 - 0.55)
	≤ ULN	0.89 (0.75 - 1.00)	0.71 (0.49 - 0.93)
Stage	Localized	0.75 (0.46 - 1.00)	0.60 (0.25 - 0.95)
	Metastatic	0.75 (0.61 - 0.89)	0.57 (0.39 - 0.75)
Visceral metastases	Yes	0.80 (0.66 - 0.94)	0.53 (0.33 - 0.73)
	No	0.66 (0.42 - 0.90)	0.66 (0.42 - 0.90)
Palliative systemic therapy	Yes	0.85 (0.73 - 0.97)	0.62 (0.44 - 0.80)
	No	0.27 (0.00 - 0.64)	0.27 (0.00 - 0.64)
